# Self-Emulsifying Formulations to Increase the Oral Bioavailability of 4,6,4′-Trimethylangelicin as a Possible Treatment for Cystic Fibrosis

**DOI:** 10.3390/pharmaceutics14091806

**Published:** 2022-08-27

**Authors:** Erica Franceschinis, Marco Roverso, Daniela Gabbia, Sara De Martin, Matteo Brusegan, Christian Vaccarin, Sara Bogialli, Adriana Chilin

**Affiliations:** 1 Department of Pharmaceutical and Pharmacological Sciences, Via Marzolo 5, 35131 Padua, Italy; 2 Department of Chemical Sciences, Via Marzolo 1, 35131 Padua, Italy; 3 Center for Radiopharmaceutical Sciences ETH-PSI-USZ, Paul Scherrer Institute, 5232 Villigen, Switzerland

**Keywords:** TMA, cystic fibrosis, SEDDS, oral bioavailability

## Abstract

4,6,4′-trimethylangelicin (TMA) is a promising pharmacological option for the treatment of cystic fibrosis (CF) due to its triple-acting behavior toward the function of the CF transmembrane conductance regulator. It is a poorly water-soluble drug, and thus it is a candidate for developing a self-emulsifying formulation (SEDDS). This study aimed to develop a SEDDS to improve the oral bioavailability of TMA. Excipients were selected on the basis of solubility studies. Polyoxyl-35 castor oil (Cremophor^®^ EL) was proposed as surfactant, diethylene glycol-monoethyl ether (Transcutol^®^ HP) as cosolvent, and a mixture of long-chainmono-,di-, and triglycerides (Maisine^®^ CC) or medium-chain triglycerides (Labrafac^TM^ lipophile) as oil phases. Different mixtures were prepared and characterized by measuring the emulsification time, drop size, and polydispersity index to identify the most promising formulation. Two formulations containing 50% surfactant (*w*/*w*), 40% cosolvent (*w*/*w*), and 10% oil (*w*/*w*) (Maisine^®^ CC or Labrafac^TM^ lipophile) were selected. The results showed that both formulations were able to self-emulsify, producing nanoemulsions with a drop size range of 20–25 nm, and in vivo pharmacokinetic studies demonstrated that they were able to significantly increase the oral bioavailability of TMA. In conclusion, SEEDS are useful tools to ameliorate the pharmacokinetic profile of TMA and could represent a strategy to improve the therapeutic management of CF.

## 1. Introduction

Cystic fibrosis (CF) is the most common rare life-shortening disease, affecting approximately 32,000 people in Europe and 85,000 individuals worldwide [1]. Cystic fibrosis is an autosomal recessive chronic disease caused by mutations in the cystic fibrosis transmembrane conductance regulator (CFTR) gene that alter its synthesis, processing, and function. CFTR is the main chloride channel expressed in the membrane of epithelial cells in the airways, intestine, pancreas, and reproductive tract [2]. Most patients ultimately develop progressive lung disease with airway mucus obstruction, bacterial infection, and inflammation. Standard therapy involves the administration of symptomatic treatments that include mucolytics to dissolve thick mucus, antibiotics to treat or prevent infections, and anti-inflammatory agents to dampen chronic inflammation. New treatments that act directly on the CFTR molecular defect are now available. Combinations of CFTR modulators (correctors and potentiators) are currently approved by FDA/EMA, such as ivacaftor (potentiator) in combination with lumacaftor or tezacaftor or tezacaftor/elexacaftor (all correctors) [3].

In 2013, the EMA designated 4,6,4′-trimethylangelicin (TMA) as an orphan drug for the treatment of CF (EU/3/13/1137) [4] due to a triple-acting behavior toward the function of the CFTR. TMA, formerly known as a photoactive agent for the treatment of psoriasis by PUVA, is equipped with three types of activity useful for CF therapy: (1) inhibition of *P. aeruginosa*-dependent IL-8 transcription in different CF-derived bronchial epithelial cells (anti-inflammatory effect); (2) potentiation of CFTR-mediated chloride transport (CFTR potentiator); and (3) rescue of F508del CFTR (CFTR corrector) [5,6]. Preliminary experiments indicate that TMA interacts with the membrane spanning domain 1 of CFTR [7,8]. Therefore, although TMA seems to be a promising tool for the treatment of CF, its use as a drug is limited by its extremely poor water solubility. In fact, its Log *p* value is 3.28 [9], which means that it belongs to class II of the BCS classification system and its oral bioavailability (BA) is limited by its low water solubility [10].

To increase the BA of APIs belonging to class II, much attention has been paid in recent years to lipid-based formulations. Lipid formulations generally consist of a drug dissolved in a mixture of two or more excipients, which may be triglyceride oils, partial glycerides, water-soluble cosolvents, surfactants, or co-surfactants [11]. The clinical utility of lipid-based formulations is demonstrated by the number of commercially available products [12,13,14], such as, for example, cyclosporin A and the two HIV protease inhibitors, ritonavir and saquinavir [15].

Several reviews on lipid-based formulations are available that focus on different aspects of lipids in drug delivery and proposing a lipid-formulation classification system. In particular, Pouton suggested a classification of lipid formulations into four different types according to their compositions [11,12,16,17].

The simplest lipid formulations are those in which the drug is dissolved in digestible oil, usually a vegetable oil or medium-chain triglycerides (lipid formulations “type I”). Although the oral bioavailability of drug delivered in triglycerides may be high, their low solvent capacity limits their use to compounds with log *p* > 4. The solvent capacity for less hydrophobic drugs can be improved by blending triglycerides with other oily excipients, including mixed monoglycerides and diglycerides. Furthermore, to promote emulsification and increase the solvent capacity, a surfactant with a hydrolipophilic balance (HLB) lower than 12 could be introduced into the formulation (lipid formulations “type II”). Hydrophilic surfactants (HLB > 12) and/or water-soluble cosolvents have also been mixed with oils to produce self-emulsifying systems (lipid formulations “Type III”). When the surfactant content is high (>40%) or co-solvents are included in addition to surfactants, it is possible to produce very fine dispersions (<100 nm in diameter) under conditions of gentle agitation. The introduction of hydrophilic surfactants and co-solvents can also increase the solvent capacity of drugs having a log *p* value in the range of 2–4 [11]. Finally, “type IV” formulations do not include an oily phase and are useful for hydrophobic but not lipophilic drugs [17]. The ability of lipid formulations to enhance the absorption of lipophilic molecules has been well-known for many years. The primary mechanism by which lipid-based formulations improve bioavailability is solubilization of the drug, although other mechanisms of absorption enhancement have been implicated, including reduction of P-glycoprotein-mediated efflux, mitigation of hepatic first-pass metabolism through improved lymphatic transport, prolongation of gastrointestinal tract residence time, increasing intestinal wall permeability, and protection from degradation in the gastrointestinal tract [13,14,18,19]. This study aimed to design a type III lipid formulation containing TMA to improve the oral bioavailability of this drug by mainly enhancing its solubility. Lipid formulations belonging to type III correspond to self-emulsifying drug delivery systems (SEDDS) that are mixtures containing drugs, an oil phase, surfactants and co-surfactant/cosolvents that spontaneously self-emulsify upon contact with the aqueous environment in the gastrointestinal tract to form a milky emulsion or a transparent emulsion with a slightly bluish appearance [16,20]. Furthermore, since the nature of the lipid affects the bioavailability of the drug by influencing its solubilization and the emulsification process, we developed and compared formulations containing two different oil phases, the first containing a mixture of monoglycerides, diglycerides, and triglycerides of long-chain fatty acids, with the latter containing medium-chain triglycerides to assess whether the effect on BA of TMA was dependent on the composition of SEDDS [21].

## 2. Materials and Methods

### 2.1. Materials

4,6,4′-Trimethylangelicin (TMA) and 6,4′-dimethylangelicin, used as internal standard (IS), were synthesized as previously reported [22]. Glycerol/Glyceryl monolinoleate (Maisine^®^ CC), medium-chain triglycerides (Labrafac^TM^ Lipophile WL1349), diethylene glycol-monoethyl ether (Transcutol^®^ HP), propylene glycol monocaprylate (Capryol^®^ PGMC), propylene glycol monolaurate (Lauroglycol^TM^ FCC), caprylocaproyl polyoxyl-8 glycerides (Labrasol^®^ ALF), were obtained from Gattefossé (Saint-Priest, France). Polyoxyl-35-castor oil (Cremophor^®^ EL) was supplied by BASF (Ludwigshafen, Germany) and polysorbate 80 (Twen 80) by Acef Spa (Fiorenzuola D’arda, Italy). Methanol and water for HPLC-HRMS analysis were purchased from Carlo Erba (Milano, Italy) All other chemicals and solvents were of analytical grade and were used without further purification.

### 2.2. Metabolic Stability with Human Liver Microsomes

The metabolic stability of TMA was assessed by incubating the compound with human liver microsomes (HLMs) (BD Bioscience, Franklin Lakes, NJ, USA), as already described [23]. Briefly, TMA was dissolved in DMSO (1 µM) and incubated at 37 °C for 10 and 30 min in 0.1 M phosphate buffer containing 200 µg of microsomes and 10 mM NADPH, pH 7.4. At the end of incubation, the reaction was stopped by addition of 200 µL cold acetonitrile. After centrifugation for 10 min at 12,000× *g* (Hettich Mikro 120 benchtop centrifuge), supernatants were collected and analyzed by means of ultra-high pressure liquid chromatography coupled to high-resolution mass spectrometry (UHPLC-HRMS) to assess metabolic stability of TMAas described in Section 2.8.1.

### 2.3. Solubility Studies

The solubility of TMA in different oils, surfactants, co-surfactants/co-solvent was determined by pouring an excess of TMA in a 2 mL glass vial containing 1 g of each excipient. The mixtures were vortexed and kept at 25 °C for 24 h, and then the samples were centrifuged at 15,000× *g* rpm for 10 min to remove the undissolved drug. The supernatant was diluted with a suitable solvent and the TMA was quantified by HPLC (Agilent 1220 Infinity LC, Agilent Technologies, Santa Clara, CA, USA) equipped with a UV-vis detector and a Zorbax 300SB C 18 column (4.6 × 250 mm, 5 µm, Agilent Technologies, Santa Clara, CA, USA). For the analysis of the organic solutions of TMA, a volume of 20 µL was injected into the column and a mixture of methanol and water (95:5) was used as mobile phase with a flow rate of 0.8 mL/min. Instead, for the analysis of aqueous solutions, 100 µL of sample was injected and a mixture 75:25 of methanol and water was used as mobile phase at a flow rate of 0.9 mL/min. The UV was set at a wavelength of 251 nm.

### 2.4. Phase Diagram Study and Determination of Self-Emulsification Time

The ternary phase diagrams of oil, surfactant, and cosolvent/cosurfactant were developed using the water titration method. A series of mixtures containing different amounts of oil (10–30%), surfactant (20–50%), and cosolvent/cosurfactant (20–50%) were prepared; 200 µL of each mixture was then diluted in 200 mL of water that was kept at 37 °C under mild agitation and visually observed to assess its ability to emulsify spontaneously. Gentle agitation was provided by a standard stainless steel dissolution paddle rotating at 50 rpm. The tendency to emulsify was judged to be good when the droplets spread easily in water and formed a fine milky emulsion. It was considered bad when there was poor or no emulsion formation. For formulations able to emulsify, the emulsification time of SEDDS was evaluated. The results are averaged from three replicated experiments.

### 2.5. Droplet Size of Microemulsion and Zeta Potential

The droplet size distribution of the diluted SEDDS was measured using a laser light scattering analyzer (Zetasizer ZS 90, Malvern Instruments, Worcestershire, UK). All SEDDS were diluted in a ratio of 1:250 (*v*/*v*) with distilled water and mixed for 1 min prior to testing. From the light scattering signal (monitored at 25 °C and 173° in automatic mode), the intensity-weighed diameter of dispersion droplets (reported as the z-average) and the polydispersity index (PDI) were calculated using the manufacturer’s software. The zeta potential of the selected formulations was determined by electrophoretic light scattering technique using the Zetasizer ZS 90 (Malvern Instruments, Worcestershire, UK) with a laser wavelength of 633 nm at a temperature of 37 °C. 

### 2.6. In Vitro Dissolution Test

In vitro drug dissolution tests were performed using the USP 24 method with a dissolution apparatus 2 (Sotax AT7 Smart, Sotax, Allschwil, Switzerland) at 100 rpm. Dissolution tests were carried out under sink conditions at 37 ± 0.5 °C in 900 mL of two simplified simulated intestinal fluids to simulate the fed and fasted states. The composition of simplified FaSSIF (*fasted-state simulated intestinal fluid*) and FeSSIF (*fed-state simulated intestinal fluid*) buffers were those reported by Zoeller and Klein [24]. Samples (1 mL) were withdrawn at 5, 10, 15, 30, 45, 60, 90, 120, 240, and 300 min and the volume removed was replaced with an equal volume of temperature-equilibrated media. The samples were filtered through a 0.45 µm acetate cellulose filter and the TMA concentration was determined by HPLC-UV using the method reported in Section 2.3. The results were averaged from three replicated experiments.

### 2.7. In Vivo Pharmacokinetics

All the experimental procedures involving animals were conducted in compliance with national and international guidelines for the handling and use of experimental animals (Authorization no. 853-2018-PR, 11 November 2018) and appropriately designed to minimize their pain or discomfort. All the animals were maintained in IVC cages under controlled conditions, with a 12/12 h dark/light cycle and free access to food and drink. For the pharmacokinetic analysis, a 20 mg/kg dose of TMA, suspended in water or dissolved in formulations A.1 and B.1, was administered to mice by oral gavage, after an overnight fast. Plasma samples (*n* = 3 per time point) were collected from the submandibular plexus before and 0.25, 0.5, 1, 2, 3, 4, 6, 24, and 48 h after the administration of TMA. The main PK parameters were calculated by means of the Microsoft Excel plug-in PK Solver, using standard formulae [25]. The data were analyzed using the software GraphPad Prism 8.0 (GraphPad Software Inc., San Diego, CA, USA) ver. 8.0, and, if not otherwise stated, are expressed as mean ± SD. Statistical analysis was performed using Student’s *t* test or one-way ANOVA, when appropriate, and *p* < 0.05 was considered statistically significant.

### 2.8. Identification of TMA-Related Metabolites in HLMs Mixture and Quantification of TMA in Plasma Samples by UHPLC-HRMS

#### 2.8.1. Stability of TMA in HLMs

The HLM reaction mixture was spiked with 80 nM of internal standard (IS, 6,4′-dimethylangelicin) [22], centrifuged for 10 min at 12,000× *g* (Hettich Mikro 120 benchtop centrifuge) and 25 °C, and directly injected into the UHPLC-HRMS system without further pretreatments.

The UHPLC-HRMS system was equipped with Agilent 1260 Infinity II LC System coupled to an Agilent 6545 LC/Q-TOF mass analyzer (Agilent Technologies, Palo Alto, Santa Clara, CA, USA). The analytical column was a Kinetex 2.6 μm C18 Polar, 100 A, 100 × 2.1 mm (Phenomenex, Bologna, Italy), thermostated at 30 °C. The components of the mobile phase A and B were water and methanol, respectively, both containing 10 mM ammonium formate. The eluent flow rate was 0.25 mL/min. The mobile phase gradient profile was as follows (t in min): t_0–3_ 2% B; t_3–18_ 2–100% B, t_18–20_ 100% B; t_20–30_ 0% B. The MS conditions were: electrospray (ESI) ionization in positive mode, gas temperature 325 °C, drying gas 10 L/min, nebulizer 20 psi, sheath gas temperature 400 °C, sheath gas flow 12 L/min, VCap 4000 V, nozzle voltage 0 V, fragmentor 180 V. Centroid full scan mass spectra were recorded in the range 100–1000 *m*/*z* with a scan rate of 1 spectrum/s. MS/MS data were acquired in targeted mode with a scan rate of 1 spectrum/s, collision energy of 35 eV, and isolation width of 4 Da. The QTOF calibration was performed daily with the manufacturer’s solution in this mass range. The MS and MS/MS data were analyzed by the Mass Hunter Qualitative Analysis software (Agilent Technologies, Palo Alto, Santa Clara, CA, USA). The chromatographic peak of TMA and related metabolites were identified and integrated by considering the extracted ion chromatogram (EIC) of the [M + H]^+^ species selected with a window of 5 ppm, and normalized by the IS area for quantification purpose. The presence of MS signals coming from potential phase 1 metabolites of TMA was simulated by the Biotransformation Mass Defects software (Agilent Technologies, Palo Alto, Santa Clara, CA, USA), and the resulting signals were searched for in the acquired chromatograms.

#### 2.8.2. TMA and TMA-Related Metabolites in Plasma Samples

TMA was extracted from the samples by treating 100 µL of plasma with 300 µL of cold acetonitrile spiked with 80 nM of IS, and then the samples were centrifuged for 10 min at 12,000× *g* (Hettich Mikro 120 benchtop centrifuge) and 25 °C. 10 μL of the supernatant were injected into the UHPLC system. The quantification of TMA was carried out by external calibration of the normalized signals using a seven-point calibration curve obtained by spiking blank plasma with TMA in the range 20–3000 nM. Linearity showed a R^2^ > 0.98 and the limit of detection of the method was assessed as about 10 nM. The metabolites related to TMA were quantified in plasma by comparing the normalized area with the TMA calibration curve as there were no suitable analytical standards available. The data obtained should be considered semiquantitative, as it is not possible to ensure the same molar response factor for TMA and its metabolites under electrospray conditions.

## 3. Results

### 3.1. Development of TMA Formulations and In Vitro Characterizations

The appropriate formulation to deliver TMA was selected based on a preformulation study that evaluated TMA solubility in different excipients (oils, surfactants, cosurfactants, and co-solvents) to identify those in which the highest drug solubility can be achieved. Results obtained from the solubility studies are reported in Table 1.

Since the nature of the lipid used affects the bioavailability of the drug by influencing its solubilization and the emulsification process [26], two oil phases were considered: the first was a mixture of monoglycerides, diglycerides, and triglycerides of long-chain fatty acids, mainly linoleic and oleic acid (Maisine^®^ CC); the latter was a mixture of medium-chain triglycerides (mainly capric and caprylic acid derivatives (Labrafac^TM^ Lipophile WL1349). The results reported in Table 1 show that TMA presents good solubility in both oils; however, since the TMA has a log *p* < 4, its solubility is higher in the mixture of monoglycerides, diglycerides, and triglycerides than in medium-chain triglycerides [11]. Instead, the appropriate cosolvent or co-surfactant, and surfactant were selected based on data solubility, and thus Transcutol^®^ HP was selected as the cosolvent and Cremophor^®^ EL (HLB 12-14) as the surfactant. 

**Figure 1:**
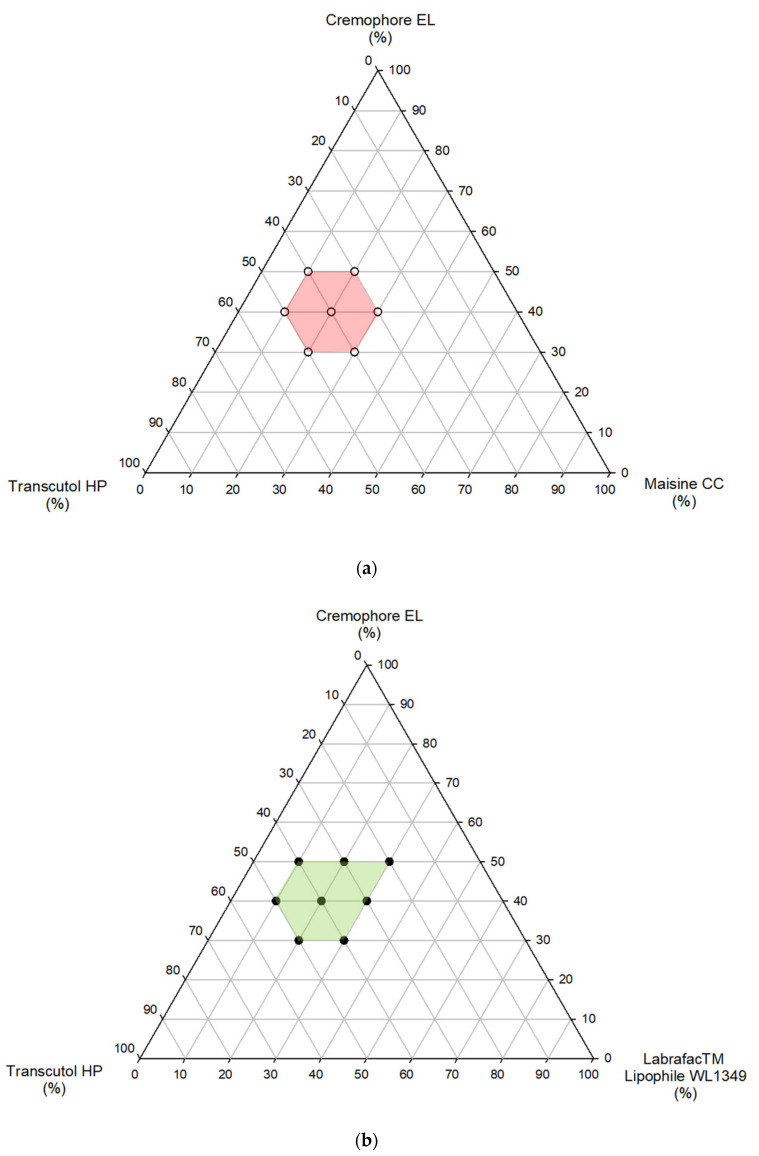
Ternary phase diagrams for formulations A, containing Maisine CC, (**a**) and formulations B, containing Labrafac Liphophile WL 1349, (**b**) as oil phases.

Afterwards, ternary phase diagrams were obtained using the water titration method to identify the mixtures containing oils (Cremophor^®^ EL and Transcutol^®^ HP) able to self-emulsify (Figure 1). A series of mixtures containing between 10 and 30% (*w*/*w*) oil, 20 and 50% (*w*/*w*) Cremophor^®^ EL, and between 20 and 50% (*w*/*w*) Transcutol^®^ HP were prepared. Each mixture was then diluted with water under mild agitation to assess the aptitude to self-emulsify. For the formulations able to self-emulsify, the time necessary for self-emulsification was evaluated and the droplet size and polydispersity index (PDI) were measured. The results are reported in Table 2 and Table 3. When the mixture is able to self-emulsify, a white, milky emulsion or a transparent emulsion is formed after dispersion; in particular, when the droplet size is less than 200 nm, a transparent emulsion with a slightly bluish appearance is produced [16].

**Figure 2:**
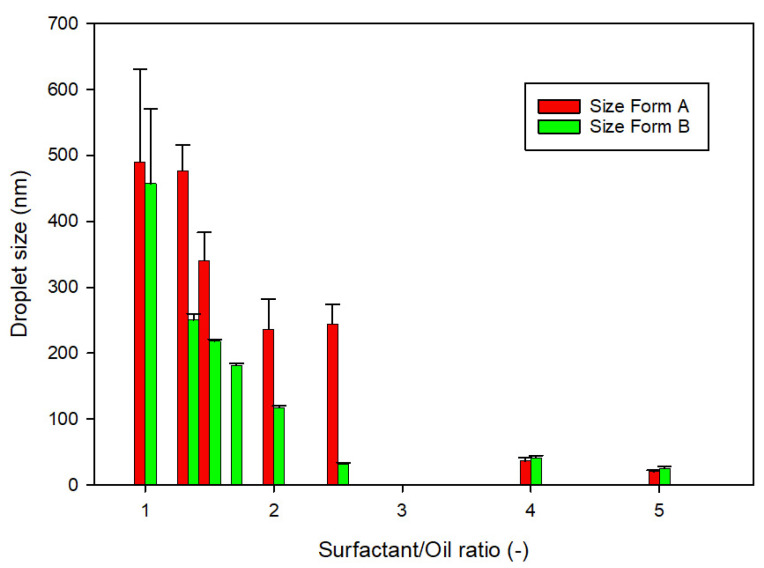
Relationship between the surfactant/oil ratio and the droplet size for formulations A, containing Maisine^®^ CC, and for formulations B, containing Labrafac^TM^ Lipophile WL1349 (*n* = 3).

Since formulations A.6, A.9, and B.9 were not able to self-emulsify, they were immediately discharged. The remaining formulations were then characterized by DLS analysis to evaluate the drop size and the PDI. Formulations with a PDI value greater than 0.7 indicate that the sample has a very broad size distribution, and thus they were dismissed. To evaluate the effect of the composition on droplet size, the ratio between the amount of surfactant and the oil versus the droplet size is reported in Figure 2. Data show that the higher the surfactant/oil ratio, the lower the droplet size.

To increase the oral bioavailability of TMA, formulations having the smallest droplet size were selected to continue the study, thus formulations A.1 and B.1, containing 10% (*w*/*w*) Maisine^®^ CC or Labrafac^TM^ Lipophile WL1349, respectively, and 50% (*w*/*w*) Cremophor^®^ EL and 40% (*w*/*w*) Transcutol^®^ HP were prepared introducing TMA to obtain formulations with a drug concentration of 4 µg/µL. Loaded formulations were characterized by measuring the droplet size, PDI, and zeta potential. The results are reported in Table 4.

**Figure 3:**
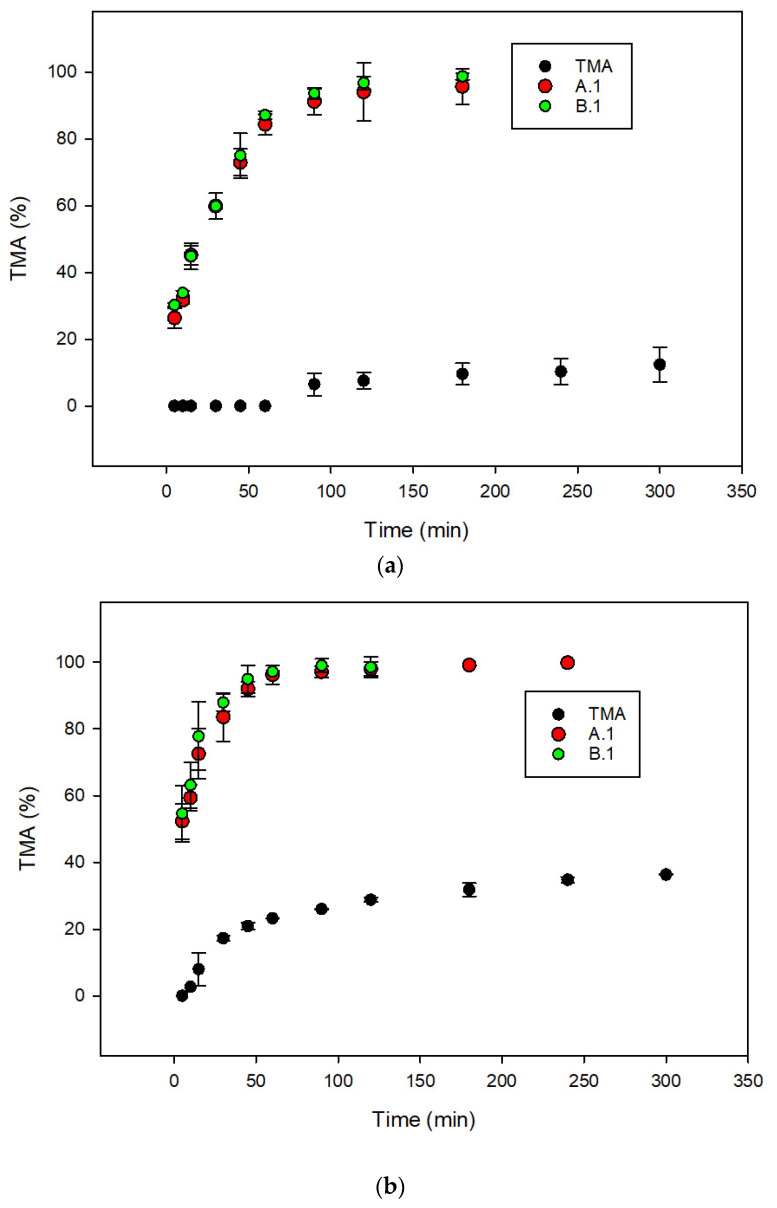
Dissolution profiles of TMA and TMA delivered in formulations A.1 and B.1 obtained using FaSSIF (**a**) and FeSSIF (**b**) buffers as dissolution media.

The results show that the introduction of the TMA did not influence the droplet size, and the PDI and both formulations present a zeta potential of approximately −9 mV. These formulations contain significant proportions of the cosolvent (Transcutol^®^ HP), and thus there is a risk that the solvent capacity of the formulation may be lost after dispersion and digestion because the cosolvent may migrate into the aqueous phase [16]. To evaluate the ability of formulations A.1 and B.1 to maintain the TMA in solution after the dispersion in aqueous fluid, dissolution tests were carried out. Furthermore, to assess the effect of the presence of food on the dissolution rate of TMA and on the performance of SEDDS formulations, two dissolution media were used: FaSSIF (fasted-state simulated intestinal fluid) and FeSSIF (fed-state simulated intestinal fluid). The dissolution profiles of free TMA and TMA in formulations A.1 and B.1 are reported in Figure 3. The results highlight that the amount of free TMA dissolved increases significantly in the presence of FeSSIF due to the presence of Tween 80 simulating the activity of bile salts produced during digestion.

### 3.2. Metabolic Stability of TMA

**Figure 4:**
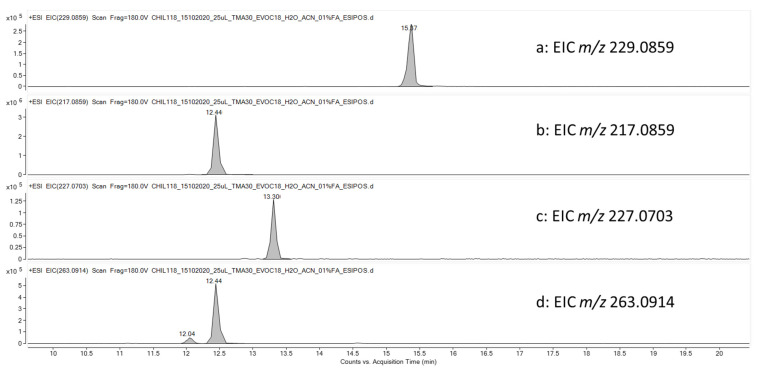
Extracted ion chromatograms (EIC) obtained after treating TMA for 30 min with human liver microsomes. (**a**) *m*/*z* 229.0859 (residual TMA, raw formula: C_14_H_12_O_3_); (**b**) *m*/*z* 217.0859 (concomitant demethylation and hydrogenation of TMA, raw formula: C_13_H_12_O_3_); (**c**) *m*/*z* 227.0703 (desaturation or hydroxylation followed by dehydration of TMA, raw formula: C_14_H_10_O_3_); (**d**) *m*/*z* 263.0914 (conversion of an alkene moiety of TMA into a diol, raw formula: C_14_H_14_O_5_).

**Figure 5:**
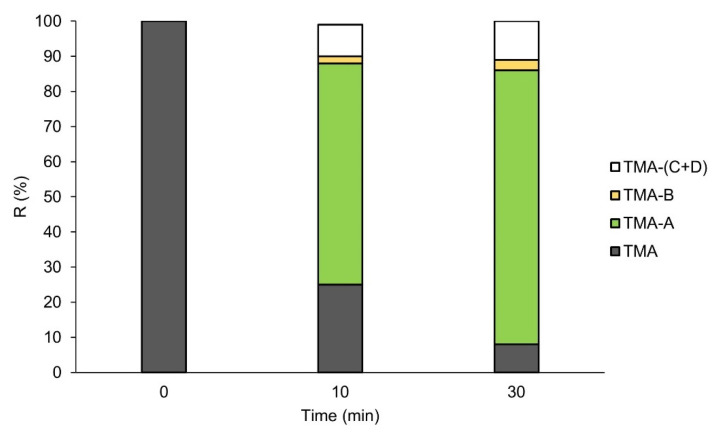
Quantitative distribution of TMA and related metabolites in the first 30 min of HLM treatment. Data are reported as relative response of each molecule with respect to the sum of the area of TMA and metabolites themselves.

To evaluate the metabolic stability of TMA, experiments using human liver microsomes (HLMs) were performed. The results highlighted a high rate of degradation for TMA after treatment with microsomal enzymes. In particular, 67 ± 15% of the initial amount of TMA was metabolized by HLM enzymes in the first 10 min, reaching a degradation of 90 ± 15% after 30 min. The potential metabolites were simulated by the Biotransformation Mass Defects software and tentatively identified by HPLC-HRMS/MS analyses. Among the simulated metabolites, three compounds generated by combining desaturation, hydroxylation, dehydration or oxidation reactions from TMA were highlighted by considering the extracted ion chromatograms of the theoretical *m*/*z* values (EIC, mass tolerance 5 ppm) and are reported in Figure 4 together with the [M+H]^+^ species (*m*/*z* 229.0859) generated by the ionization of TMA, present as a peak at 15.87 min (Figure 4a). Figure 4b reports the EIC for *m*/*z* 217.0859 (TMA-A) at 12.44 min that is putatively due to the concomitant demethylation and hydrogenation of TMA. The peak at 13.31 min at *m*/*z* 227.0703 (TMA-B) was assigned to a metabolite originated by desaturation or hydroxylation followed by dehydration of TMA (Figure 4c). The EIC at *m*/*z* 263.0914 resulted in two peaks at 12.04 (TMA-C) and 12.44 min (TMA-D) (Figure 4d) that are attributed to the conversion of an alkene moiety into a diol, occurring in two different positions of the TMA structure. The two signals, chromatographically resolved under experimental conditions and having different intensities, support the hypothesis that two different structural isomers were formed, with different probability/efficiency. The MS/MS fragmentation spectra of the highlighted metabolites (Figure S1), and although they provided the selectivity and accuracy able to empower these assignments, they were not informative for determining the correct positions of the proposed modifications to gain the definitive structures of the metabolites. Considering potential different molar response factors among TMA and related metabolites, a preliminary evaluation of the extent of degradation/production rates is possible by considering the relative response of each molecule with respect to the sum of the area of TMA and metabolites themselves (Figure 5). The highest production rate was observed for *m*/*z* 217 (TMA-A), which is to be considered the main metabolites of TMA. Lower production rates were obtained for the two isomers at *m*/*z* 263 (TMA-(C+D)) and *m*/*z* 227 (TMA-B), respectively.

### 3.3. In Vivo Pharmacokinetics of TMA

**Figure 6:**
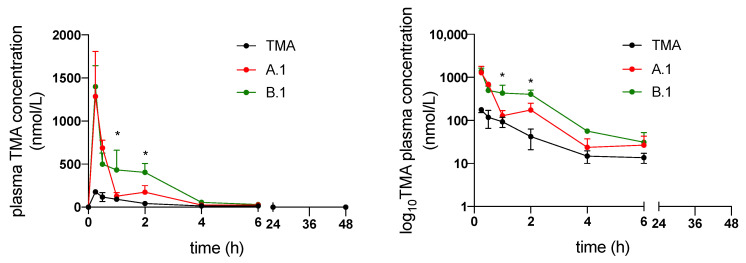
Plasma concentration vs. time curves of TMA suspended in water (black) and solubilized in formulations A.1 (red) and B.1 (green) (*n* = 3, data are presented as mean ± S.E.M.). The data are reported in a linear (left) and semi-logarithmic (right) graphs. * *p* < 0.05 vs. A.1.

Figure 6 shows the plasma concentration versus time curves obtained after the administration of TMA suspended in water and solubilized in formulations A.1 and B.1. Plasma profiles clearly indicate that both A.1 and B.1 formulations produced a dramatic increase in TMA bioavailability. In detail, A.1 led to a 3.5-fold increase and B.1 to a 5.7-fold increase in TMA bioavailability. This is probably because B.1 caused a prolongation of TMA absorption, as indicated by the plasma concentrations of TMA formulated in B.1, which are significantly higher than those of TMA formulated in A.1 1 and 2 h after administration.

The analysis of the pharmacokinetic parameters clearly reflects the plasma profiles of TMA, since both AUC and C_max_ increased significantly when TMA is formulated in A.1 and B.1, confirming the effect of the two formulations on oral bioavailability of the drug (Table 5). Accordingly, the apparent oral clearance (CL/F) and the apparent volume of distribution (Vz/F) are significantly decreased due to the increase of bioavailability (F). The elimination half-life (t_1/2_) is not affected by SEDDS. 

**Figure 7:**
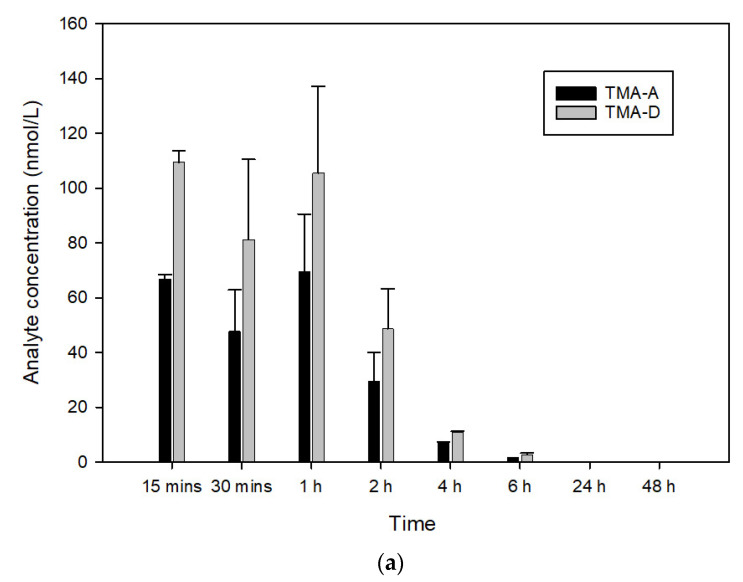

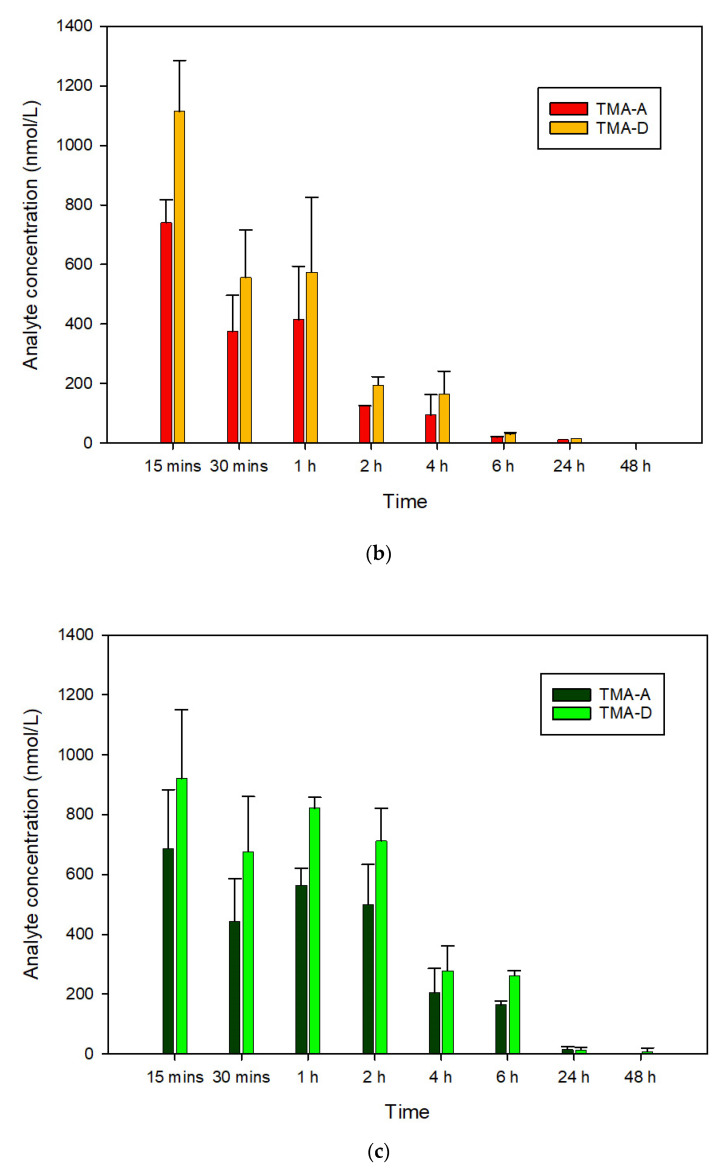
Plasma concentration versus time curves of TMA-related metabolites, TMA-A and TMA-D, considering TMA suspended in water (**a**), solubilized in formulations A.1 (**b**), and B.1 (**c**) (*n* = 3, data are presented as mean ± S.D.).

Taking into account the high rate of degradation of TMA by HLM enzymes (90 ± 15% after 30 min) and the production of the described by-products (Figure 5), further evaluations were performed in mouse plasma samples to verify the presence of the metabolites evidenced in the metabolic stability study. Their putative concentration (Figure 7) was calculated by comparing their area normalized with the calibration curve of TMA, hypothesizing the same molar response factor for all the molecules. Interestingly, the in vivo mouse study, in addition to confirming the low metabolic stability of TMA (Figure 5), showed differences in the metabolite production since TMA-D was found to be the main metabolite, while TMA-A had a lower production rate compared to the HLM study; furthermore, TMA-B and TMA-C were lower than LOD. This can be explained by a potential different expression and activity of murine hepatic enzymes compared to HLM, which could affect the production rate of the different TMA metabolites.

## 4. Discussion

TMA is a very interesting triple-acting agent for the treatment of CF, but its use in clinics is limited by its low oral bioavailability due to limited water solubility and low metabolic stability. Among the different approaches that can be proposed to increase the water solubility of poorly water-soluble compounds in recent years, much attention has been focused on lipid-based formulations, especially self-emulsifying drug delivery systems (SEDDS) [12]. Recently, Ditzinger et al. analyzed some physicochemical properties of drugs already approved in lipid-based formulations by the FDA, observing that most of these active molecules have a melting point lower than 230 °C, a molecular weight in the range of 200–500 g/mol, and a log *p* value between 0.8 and 7.5 [27]. Since TMA has a molecular weight of 228 g/mol, a log *p* of 3.28, and a melting temperature of 201.83 °C, it could represent a good candidate to develop a successful lipid formulation. Consequently, this study aimed to design a SEDDS-based TMA formulation capable of self-emulsifying in the presence of gastrointestinal fluids, leading to increased oral bioavailability of TMA. Since the nature of lipid used affects the bioavailability of the drug by influencing its solubilization and emulsification process [26], two different oil phases were considered in this study: the first was Maisine^®^ CC (glyceryl monolinoleate) a mixture of monoglycerides, diglycerides, and triglycerides (mainly linoleic and oleic acid) that represents a digestible long-chain glyceride with a good solvent property and capable of promoting lymphatic absorption; the second was Labrafac^TM^ Lipophile WL1349, a mixture of medium-chain triglycerides (mainly capric and caprylic acid derivatives) [21]. These two oil phases differ in the length of the fatty acids and in the number of free hydroxyl groups, producing differences in surface activities and polarity. In addition, the in vivo digestion process is also different, and this could affect the solubility of the drug in the gastro-intestinal fluids [12,28].

TMA showed a good solubility in both oils and, as expected, its solubility was higher in the mixture of monoglycerides, diglycerides, and triglycerides (Maisine^®^ CC) than in medium-chain triglycerides (Labrafac^TM^ Lipophile WL1349) since TMA has a log *p* < 4 [11]. Among the surfactants tested, Cremophor^®^ EL (HLB 12-14), a polyoxyl castor oil derivative, was selected because the drug solubility was higher compared to other tested products. Furthermore, it was reported that Cremophor^®^ EL (HLB 12-14) enhances the intestinal absorption of drugs by promoting the opening of the tight junctions and inhibiting P-glycoprotein [26,29]. Since SEDDS belong to class II and III lipid formulations, a hydrophilic cosolvent and/or a water insoluble surfactant (co-surfactant) are needed in the formulation to increase drug solubilization, decrease the droplet size of the microemulsion, and improve the ability to self-emulsify [17]. The hydrophilic cosolvent Transcutol^®^ HP was selected due to the higher TMA solubility and for its ability to inhibit the P-glycoprotein [30].

The two formulations with the smallest droplet size (A.1 and B.1) were selected for further evaluation as a large surface area facilitates the enzymatic breakdown of lipid droplets by pancreatic lipase. Therefore, the droplet size of the SEDDS after dispersion are implied in the fate of the formulation after ingestion, and a smaller droplet size allows a faster and more reproducible drug release compared with simple oil solutions. Furthermore, an improved solubilization of lipophilic drugs can be mediated by the generation of (mixed) micelles and various colloidal structures from the interaction of lipid digestion products with endogenous bile salts and phospholipids [31]. Bile salts act as wetting and solubilizing agents, thus promoting the dissolution and the absorption of poorly water-soluble molecules. Both the selected SEDDS formulations can rapidly disperse in aqueous media and maintain the TMA in dissolution form. However, the dispersion of both formulations in FeSSIF is faster (t_50_ of about 5 min versus t_50_ of 15 min), probably because the presence of surfactant facilitates the dispersion of the self-emulsifying formulations. To verify the ability of the self-emulsifying systems formulated to increase the oral bioavailability of TMA and to evaluate any absorption differences related to the nature of the two oily phases, an in vivo pharmacokinetic study was performed. This study demonstrated a dramatic increase in BA of TMA when formulated in both A.1 and B.1. These results agree with findings by Pouton [11]. In fact, the oral bioavailability from oil solutions is likely to be optimal because the triglycerides are digested to free fatty acids and 2-monoglycerides, and these products are solubilized to form a colloidal dispersion within bile salt-lecithin-mixed micelles. As consequence, the drug could be further solubilized in mixed micelles resulting in a reservoir of the drug in colloidal solution available for a passive (transcellular) absorption. We can speculate that the increased bioavailability may not be due to a reduction of hepatic first-pass, although we demonstrated that TMA is characterized by poor metabolic stability because B.1 (enhancing intestinal absorption) caused a higher increase in BA than A1 (facilitating lymphatic absorption). Therefore, the prolonged absorption observed for the B.1 formulation probably leads to an increase of TMA that reaches the liver and then the systemic circulation, indicating that an enhancement of intestinal absorption is more effective than the possible reduction of hepatic first-pass for the increase in TMA bioavailability. Furthermore, the plasma concentration of TMA metabolites was similar with A.1 and B.1, indicating that the extent of hepatic metabolism was not affected by the formulation. However, further mechanistic studies are necessary to ascertain the exact mechanism by which these self-emulsifying formulations cause a differential effect on the increase of TMA bioavailability. Interestingly, the elimination half-life (t_1/2_) is not affected by SEDDS, confirming that A.1 and B.1 act on TMA bioavailability without affecting its systemic pharmacokinetic processes. Furthermore, two of the four TMA metabolites identified in the HLM mixture were detectable also in mouse plasma (Figure 7), and their concentration confirmed that the main mechanism by which SEDDS increase the BA of TMA is the enhancement of intestinal absorption.

## 5. Conclusions

In this study, we developed a SEDDS formulation based on polyoxyl-35 castor oil (Cremophor^®^ EL) as surfactant, diethylene glycol-monoethyl ether (Transcutol^®^ HP) as cosolvent and glycerol/glyceryl monolinoleate, and monoglycerides, diglycerides, and triglycerides (Maisine^®^ CC) or medium-chain triglycerides (LabrafacTM lipophile WL1349) to increase oral bioavailability of TMA. Both formulations led to an increase in TMA solubility and a fast dispersion of droplet in the size range of 20–25 nm and were able to produce a significant increase of the oral TMA bioavailability in vivo. This effect is probably due to an enhanced absorption of the drug by helping the production of mixed micelles representing a drug reservoir available for absorption. In conclusion, SEDDS are useful tools to improve the pharmacokinetic profile of TMA and represent a smart strategy to improve the therapeutic management of cystic fibrosis.

## Figures and Tables

**Table 1:** Screened excipients, their role and characteristics, and solubility of TMA (*n* = 3).

Name	Role	Chemical Name	HLB	TMA Solubility (µg/mL)
Maisine^®^ CC	Oil	Glycerol/Glyceryl monolinoleate	1	6340 ± 377
Labrafac^TM^ Lipophile WL1349		Medium chain triglycerides	1	4639 ± 170
Transcutol^®^ HP	Co-solvent	Diethylene glycol-monoethyl ether	-	9568 ± 131
Ethanol		Ethyl alcohol	-	2509 ± 52
Capryol^®^ PGMC	Co-surfactant	Propylene glycol monocaprylate	6	7346 ± 380
Lauroglycol^TM^ FCC		Propylene glycol monolaurate	5	6484 ± 92
Cremophor^®^ EL	Surfactant	Polyoxyl-35-castor oil	12–14	9091 ± 217
Tween 80		Polysorbate 80	15	7040 ± 120

**Table 2:** Composition of formulations containing Maisine^®^ CC as oil phase, their self-emulsification time, droplet size, and PDI (*n* = 3).

Formulation		Maisine^®^ CC (% *w*/*w*)	Cremophor^®^ EL (% *w*/*w*)	Transcutol^®^ HP (% *w*/*w*)	Self-Emulsification Time (s)	Droplet Size (nm)	PDI (-)
A	1	10	50	40	7 ± 1	20.37 ± 1.76	0.19
	2	10	40	50	7 ± 1	36.29 ± 5.30	0.20
	3	20	50	30	8 ± 1	243.96 ± 29.71	0.29
	4	20	40	40	9 ± 2	236.30 ± 45.31	0.51
	5	20	30	50	9 ± 1	341.03 ± 42.66	0.48
	6	30	50	20	-	-	-
	7	30	40	30	39 ± 6	477.30 ± 38.40	0.52
	8	30	30	40	17 ± 4	490.31 ± 140.52	0.82
	9	30	20	50	-	-	-

**Table 3:** Composition of formulations containing Labrafac^TM^ Lipophile WL1349 as oil phase, their self-emulsification time, droplet size, and PDI (*n* = 3).

Formulation		Labrafac^TM^ Lipophile WL1349 (% *w*/*w*)	Cremophor^®^ EL (% *w*/*w*)	Transcutol^®^ HP (% *w*/*w*)	Self-Emulsification Time (s)	Droplet Size (nm)	PDI (-)
B	1	10	50	40	8 ± 3	25.37 ± 2.71	0.16
	2	10	40	50	6 ± 2	41.32 ± 2.90	0.17
	3	20	50	30	11 ± 2	31.98 ± 1.71	0.21
	4	20	40	40	8 ± 1	117.73 ± 2.06	0.18
	5	20	30	50	8 ± 1	218.57 ± 2.61	0.19
	6	30	50	20	>90	181.47 ± 2.91	0.25
	7	30	40	30	8 ± 1	250.33 ± 9.29	0.10
	8	30	30	40	10 ± 3	457.67 ± 113.07	0.8
	9	30	20	50	-	-	-

**Table 4:** Composition of the formulations selected to continue the study, their droplet size, and PDI (*n* = 3).

Formulation	Oil (% *w*/*w*)	Cremophor^®^ EL (% *w*/*w*)	Transcutol^®^ HP (% *w*/*w*)	Droplet Size (nm)	PDI (-)	Zeta Potential (mV)
A.1	10	50	40	22.80 ± 1.80	0.25	−8.82 ± 1.84
B.1	10	50	40	23.38 ± 0.61	0.11	−9.03 ± 3.66

**Table 5:** Main pharmacokinetic parameters of TMA suspended in water (first column) or formulated with A.1 or B.1, observed or calculated from the concentration versus time curves.

Parameter (Mean ± S.D.)	Formulation		
	TMA	A.1	B.1
C_max_ (nmol/L)	186.05 ± 21.14	1285.85 ± 738.43 *	1399.60 ± 342.81 *
T_max_ (h)	0.38 ± 0.18	0.25 ± 0.00	0.25 ± 0.00
AUC_0-inf_ (nmol/L * h)	297.83 ± 119.13	1059.39 ± 90.80 *	1686.53 ± 504.57 **
t_1/2_ (h)	1.71 ± 0.16	1.09 ± 0.39	1.27 ± 0.80
Vz/F (mL/g)	0.183 ± 0.089	0.029 ± 0.008 *	0.025 ± 0.004 *
CL/F (mL/h/g)	0.073 ± 0.029	0.019 ± 0.002 *	0.012 ± 0.004 *

* *p* < 0.05, ** *p* < 0.01 vs. TMA.

## Data Availability

The data presented in this study are available in this article “Self-Emulsifying Formulations to Increase the Oral Bioavailability of 4,6,4′-Trimethylangelicin as a Possible Treatment for Cystic Fibrosis” and in Supplementary material “Figure S1”.

## Supplementary Materials

The following supporting information can be downloaded at: https://www.mdpi.com/article/10.3390/pharmaceutics14091806/s1. Figure S1: MS/Ms spectra of: (a) TMA (parent *m*/*z* 229.0859), (b) TMA-A (parent m/z 217.0859), (c) TMA-B (parent m/z 227.0703), (d) TMA-D (parent *m*/*z* 263.0914).
