# Supplementary figures and images for: Self-Emulsifying Formulations to Increase the Oral Bioavailability of 4,6,4′-Trimethylangelicin as a Possible Treatment for Cystic Fibrosis
Source: Pharmaceutics. 2022 Aug 27;14(9):1806. doi: 10.3390/pharmaceutics14091806 (PMC9506254; doi:10.3390/pharmaceutics14091806)

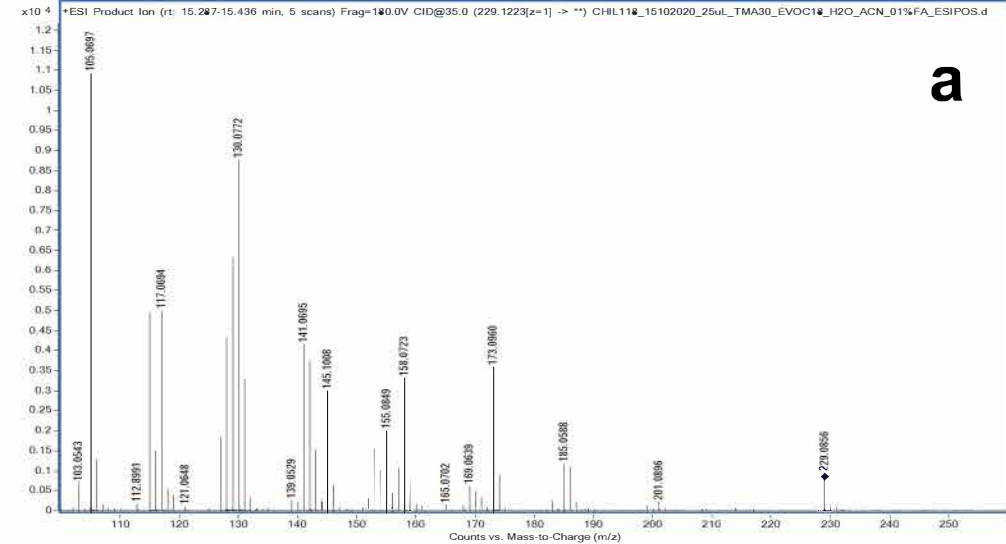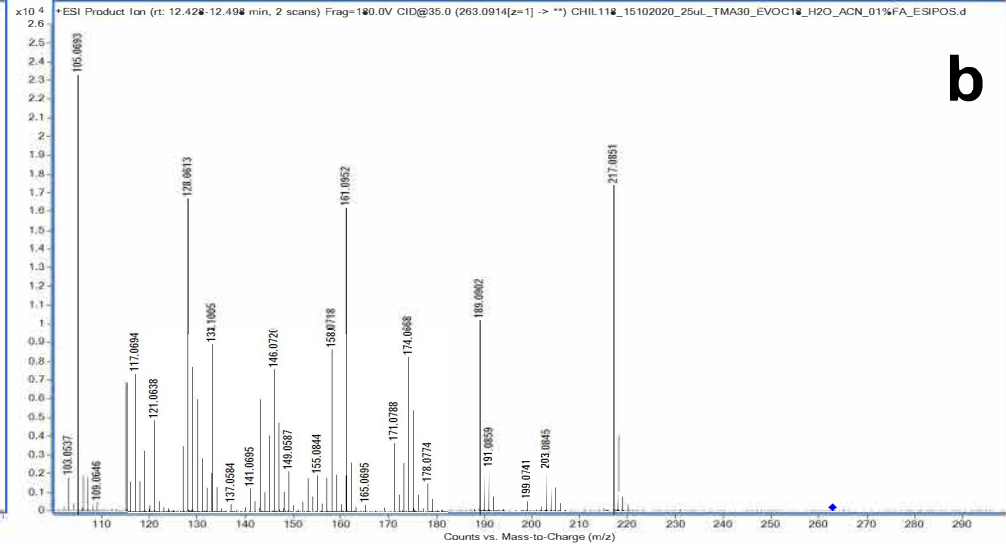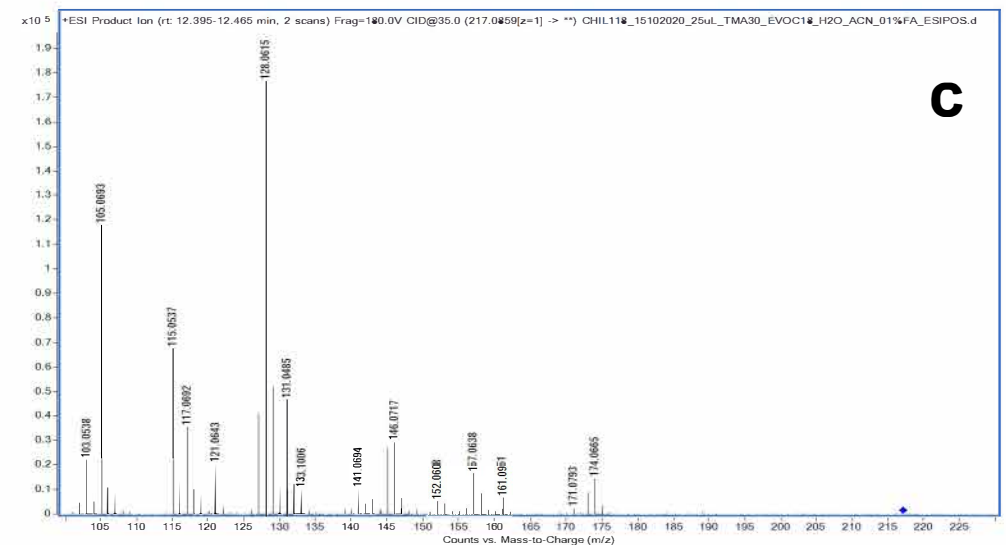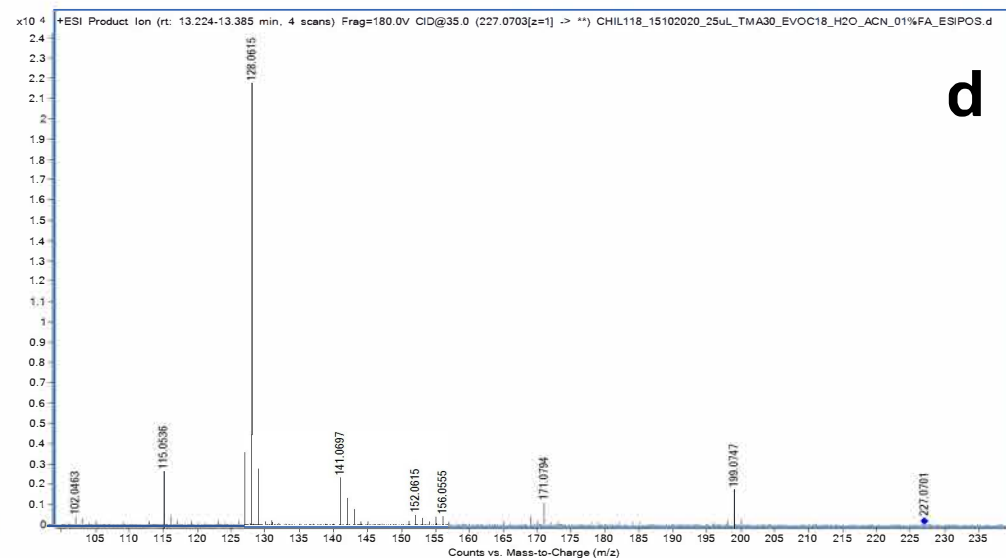

Figure S1: MS/Ms spectra of: (a) TMA (parent  $m/z$  229.0859), (b) TMA-A (parent  $m/z$  217.0859), (c) TMA-B (parent  $m/z$  227.0703), (d) TMA-D (parent  $m/z$  263.0914).

Supplement: Supplementary file 1 [file pharmaceutics-14-01806-s001.zip › pharmaceutics-1874717-supplementary.pdf]
